# Commander Blount

**Published:** 1897-06-05

**Authors:** 


### OBITUARY.

### COMMANDER BLOUNT, R.N.

By the unexpected and deeply regretted death of Commander Blount, R.N., who for nearly twenty-two years has fulfilled the duties of secretary to the Victoria Hospital for Children, Chelsea, that institution has suffered an irreparable loss. As everyone may guess, the arrangements for the huge fete to be held this month in the Royal Botanic Gardens in aid of the Victoria Hospital have meant an immense increase of work, the burden of which has been mainly if not entirely upon Captain Blount's shoulders, and it can hardly be doubted that the extra work entailed was the precipitating cause of the paralysis which attacked him on April 5th. He recovered from the paralysis. but never really rallied, dying on Wednesday, May 26th, at his home, Baldry Gardens, Streatham Common. The funeral took place very quietly at Tooting Cemetery on Monday, During all the years which Commander Blount has been connected with the Victoria Hospital he has identified himself heart and soul with its every interest, and has worked in its cause with untiring energy and marvellous success. There is something pathetic in the coming of the end just before the great event of the fete, on the success of which his heart was set, and for which he had been labouring for many months past. Commander Blount was only fifty-four years of age. He was a son of the late Commander W. S. Blount, R.N., and grandson of Captain Clavell, R.N., who was present and was wounded at the Battle of Trafalgar. He joined the navy in 1856, and left the service finally in 1873, his last command being that of the "Tyrian."

